# Use of smokeless tobacco among groups of Pakistani medical students – a cross sectional study

**DOI:** 10.1186/1471-2458-7-231

**Published:** 2007-09-03

**Authors:** Sardar Z Imam, Haq Nawaz, Yasir J Sepah, Aqueel H Pabaney, Mahwish Ilyas, Shehzad Ghaffar

**Affiliations:** 1 Department of Surgery (ENT, Head & Neck Division), Aga Khan University Medical College, Karachi, Pakistan

## Abstract

**Background:**

Use of smokeless tobacco is common in South Asia. Tobacco is a major preventable cause of morbidity and mortality. Doctors make one of the best avenues to influence patients' tobacco use. However, medical students addicted to tobacco are likely to retain this habit as physicians and are unlikely to counsel patients against using tobacco. With this background, this study was conducted with the objective of determining the prevalence of smokeless tobacco among Pakistani medical students.

**Methods:**

A cross sectional study was carried out in three medical colleges of Pakistan – one from the north and two from the southern region. 1025 students selected by convenient sampling completed a peer reviewed, pre-tested, self-administered questionnaire. Questions were asked regarding lifetime use (at least once or twice in their life), current use (at least once is the last 30 days), and established use (more than 100 times in their life) of smokeless tobacco. Chi square and logistic regression analyses were used.

**Results:**

Two hundred and twenty (21.5%) students had used tobacco in some form (smoked or smokeless) in their lifetime. Sixty six (6.4%) students were lifetime users of smokeless tobacco. Thirteen (1.3%) were daily users while 18 (1.8%) fulfilled the criterion for established users. Niswar was the most commonly used form of smokeless tobacco followed by paan and nass. Most naswar users belonged to NWFP while most paan users studied in Karachi. On univariate analysis, lifetime use of smokeless tobacco showed significant associations with the use of cigarettes, student gender (M > F), student residence (boarders > day scholars) and location of the College (NWFP > Karachi). Multivariate analysis showed independent association of lifetime use of smokeless tobacco with concomitant cigarette smoking, student gender and location of the medical college.

**Conclusion:**

The use of smokeless tobacco among medical students cannot be ignored. The governments should add the goal of eliminating smokeless tobacco to existing drives against cigarette smoking. Drives in Karachi should focus more on eliminating paan usage while those in NWFP should focus more on the use of naswar. Medical colleges should provide greater education about the myths and hazards of smokeless tobacco.

## Background

Tobacco can be used in ways ranging from cigarette, cigar and bidi smoking, to chewing of 'smokeless tobacco'. This latter category includes various forms of tobacco with paan/betel quid being the most common one used [1]. Other forms include naswar, gutka, qiwam, minpuri and other less known products. The exact compositions of these forms vary according to regional preferences. Most people place these in the mandibular or labial groove and suck on them slowly for 10–15 minutes or simply apply them to their teeth and gums [1].

Use of smokeless tobacco is integral to the culture of South Asia. Smokeless tobacco users in India and Pakistan together have been estimated to number 100 million [2]. In India about 35–40% [1] of tobacco consumption is in smokeless forms while an earlier study in Pakistan showed that 21% of men and 12% of women were users of betel quid [3]. Moreover, increasing use is being reported among vulnerable groups such as children, adolescents, women and also immigrants of South Asian descent wherever they have settled [1,4].

Factors that continue to encourage people to use smokeless tobacco include its affordability, ease of purchase or production and the widely held misconception that it has medicinal value for improvement in tooth ache, headache and stomach ache [1]. Furthermore, in contrast to cigarettes, there is no taboo against using smokeless tobacco [1] and the government efforts have also focused more on eliminating cigarette use than tobacco as a whole [1,5]. All these, coupled with peer pressure and the belief that smokeless tobacco is less hazardous than cigarette smoking mean that these forms continue to be used by vast numbers of people.

Presently tobacco use is the leading preventable cause of death globally [6] and it is estimated that by 2030, it would account for over 10 million annual deaths worldwide [7,8], 70% of which will be in the developing world [9]. All forms of tobacco carry serious health consequences, most importantly oral and pharyngeal cancers [1,10-13] and other malignancies of the upper aerodigestive tract [1,7,14]. Other ingredients combine with tobacco to produce a product with an even higher carcinogenicity for humans. Tobacco-related cancers account for about one-third of all cancers in South Asia [1] while the emerging 'epidemic' of oral submucous fibrosis [1,13] has been attributed to chewing of areca nut and its mixtures. There is also evidence that smokeless tobacco is a risk factor for hypertension and dyslipidemias [1]. Chewing of tobacco by pregnant mothers has been found to cause an increased incidence of still births and low birth weight deliveries [1]. In addition, chewing of betel quid, with or without tobacco can aggravate asthma and predispose the users to diabetes mellitus [1].

It is evident that costs and consequences of tobacco use impose a heavy social and economic burden on a nation. Much of this can be avoided by policies and awareness programmes aimed at reducing tobacco use. Doctors make one of the best avenues of such education to people and have immense potential to influence patients' tobacco use. However, doctors who themselves use smokeless tobacco are unlikely to counsel patients against using tobacco [1]. Thus assessment of the use of smokeless tobacco by medical students becomes important as students addicted to tobacco use are expected to continue the habit into their years as practicing physicians and care givers. Such data is also likely to help the colleges in assessing their curricula with regard to education on important medico-social issues like the use of tobacco. With this background, this study was conducted with the objective of determining the prevalence of smokeless tobacco among Pakistani medical students from three different medical colleges.

## Methods

This was a multi-center cross sectional study carried out on students of three medical colleges of Pakistan during the period June-August 2005. One college was selected from the north of Pakistan (province of N.W.F.P.) while two were selected from the south of the country (coastal city of Karachi) in order to study and compare the patterns of tobacco use in students from different backgrounds. The combined student strength of the three colleges was approximately 3000.

A sample size of at least 940 was required to estimate the prevalence of lifetime use of smokeless tobacco among medical students, assuming the 20 percent prevalence figure of the Pakistani population, along with 80 percent power, 0.05 significance level, 2.5 percent bond on error, and adjustment for non-response rate. We ended up approaching 1092 students out of which 1025 subjects filled the questionnaire while 67 refused consent (response rate = 93.9%). Convenience sampling was used by getting the questionnaires filled during regular college hours from students at four locations in the medical college – the lecture halls, laboratories, library and canteen. Questionnaires were filled on consecutive days until the required sample size was achieved.

A peer reviewed, pilot tested, anonymous self-administered questionnaire was used. Questions were asked regarding lifetime, current, and established use of smokeless tobacco. Lifetime users were defined as having used smokeless tobacco at least once or twice in their life. Current users were defined as having used smokeless tobacco at least once in the last 30 days while established users were defined as having used smokeless tobacco on more than 100 occasions in their lifetime. Questions were also asked regarding the form of smokeless tobacco they used, any cigarette smoking, as well as the age at which they took up these habits. Relevant demographic information was also obtained.

Ethical approval for the study was obtained from the review committee of the Center for Health Research, Lahore. Questionnaires were collected back in an unmarked envelope to ensure complete confidentiality. The study was conducted in compliance with the *'Ethical Principles for Medical Research involving Human Subjects' *of Helsinki Declaration [15]. Verbal informed consent was obtained from all subjects and documented in the presence of a witness.

Data was entered and analyzed with Statistical Package for Social Sciences (SPSS) version 13.0. Descriptive statistics of socio-demographic information and use of chewable tobacco products were obtained. Univariate and multivariate odds ratio with 95 percent confidence interval were obtained using Chi square and logistic regression analyses respectively. For all purposes, a p value of < 0.05 was considered to be significant.

## Results

Of the 1025 students that completed the questionnaire, 455 (44.4%) were males and 570 (55.6%) were females. The mean age of the sample was 21 years (SD: 1.90).

Two hundred and twenty (21.5%) students had used tobacco in some form (smoked or smokeless) in their lifetime. Sixty six (6.4%) students were lifetime users of smokeless tobacco. Thirteen (1.3%) of the students were daily users while 18 (1.8%) fulfilled the criterion for established users. Thirty one (3%) students were current occasional users of smokeless tobacco (less than daily use in the last 30 days). The frequency and form of smokeless tobacco use is shown in Table 1.

**Table 1:** Pattern of use of smokeless tobacco among medical students

		**Prevalence [%], (N)**
**Frequency of use **	**Lifetime**	6.4, (66)
	**Daily**	1.3, (13)
	**Occasionally**	3.0, (31)
	**Established**	1.8, (18)

		**Prevalence [%] of lifetime use**

**Tobacco form**	**Naswar**	3.2
	**Paan**	2.0
	**Nass**	0.6
	**Others**	0.6

	**Total**	**6.4**

Niswar (3.2%) was the most commonly used form of smokeless tobacco followed by Paan (2.0%) and nass (0.6%). Gutka, minpuri, qiwam and other forms of tobacco were less commonly used. 87.9% of naswar users belonged to NWFP while 80% of paan users studied in Karachi.

39.4% of lifetime users also smoked cigarettes while among people who had never used smokeless tobacco, only 8.3% were smokers (p value: < 10^-15^). The mean age at which the students began smoking, was 17.27 years while the mean age at which they began using smokeless tobacco was 17.30 years.

Lifetime use of smokeless tobacco was also found to have significant associations with student gender (M > F, p value: < 10^-7^), student residence (boarders > day scholars, p value: 0.02) and location of the College (NWFP > Karachi, p: < 10^-6^). The prevalence of lifetime users in different socio-demographic groups is shown in Table 2.

**Table 2:** Lifetime use of smokeless tobacco in different socio-demographic groups

		**Prevalence [%] of lifetime smokeless tobacco use (N)**
**Gender**	**Male**	10.8 (49)
	**Female**	3.0 (17)
**College**	**N.W.F.P.**	11.1 (40)
	**Karachi**	3.9 (26)
**Residence**	**Hostel**	9.2 (28)
	**Day scholar**	5.3 (38)

Multivariate analysis showed that there was a higher prevalence of smoking among students who were lifetime users versus those who had not used smokeless tobacco (O.R: 4.203 [2.279–7.751], p value: < 10^-6^). This association was independent of age, gender, residence at the hostel and location of the college.

Gender was also found to be independently associated with lifetime use of smokeless tobacco. Male students were more likely to be lifetime users than female students. (O.R: 2.198 [1.177–4.102], p value: 0.013).

An independent association was also found between lifetime use of smokeless tobacco and the location of the medical college. There was a higher prevalence of lifetime users among students from the college located in NWFP compared to Karachi. (O.R: 2.155 [1.250–3.716], p value: < 0.006). Results of multivariate analysis are shown in Table 3.

**Table 3:** Predictors of lifetime use of smokeless tobacco on multivariate analysis

**Predictor**	**O.R.**	**C.I.**	**p-value**
**Gender**	2.198	1.77 – 4.102	0.013
**Location of College**	2.155	1.250 – 3.716	<0.006
**Cigarette smoking**	4.203	2.279 – 7.751	<10^-6^

O.R., Odds Ratio; C.I., Confidence Interval.

## Discussion

Almost all studies carried out in Pakistan have focused on the patterns of cigarette smoking alone. Studies on the use of smokeless tobacco have mostly been carried out by investigators attempting to prove its association with cancers of the oral cavity and pharynx [12,16]. A study carried out in Multan [17] to find an association between bladder carcinoma in women and use of smokeless tobacco found that 47% of patients were lifetime users. Another study investigating use of tobacco among patients with peptic ulcer disease found that 23% of the patients were lifetime users [18]. Understandably, our figure of 6.6% prevalence is much lower compared to the rates among patients with conditions likely to be the result of long term use of smokeless tobacco.

Only two studies have been carried out on the use of smokeless tobacco in the general population. A study published in 1982 from a population in Karachi [3], reported that 21% of the people used betel quid (paan), but the study made no distinction between non-tobacco and tobacco-laden paan consumption. A recent study from a Karachi squatter settlement reported a 40 percent prevalence of use of smokeless tobacco [19]. Various studies in Pakistan [19,20] and India [21,22] have shown that the use of smokeless tobacco is inversely associated with the level of education and this might explain the lower prevalence reported by our study since our population comprised medical students who were also likely to be more aware of the hazards of smokeless tobacco than a common man. Higher rates of tobacco use have been reported from rural areas [23]. This may also have contributed to our lower figure since all three medical colleges were located in major urban centres of Pakistan.

Most studies, especially in India have reported paan to be the most common form of smokeless tobacco used although some in Pakistan report naswar as the more popular choice. Our study reports naswar to be the most commonly used among medical students. More significantly, it was seen that 87.9% of naswar users belonged to NWFP while 80% of paan users were from Karachi. This is because people in NWFP have cultural practices and preferences similar to those of Central Asia and Afghanistan, where naswar is in common usage [1,7,24]. On the other hand, since many families in Karachi are actually migrants from India, the use of paan is much more common in colleges of Karachi, a trend that resembles neighboring India [1,24,25].

We also report an independent association between the use of smokeless tobacco and the location of the college, with students from NWFP being more likely to be lifetime users. One explanation of this is the relative racial homogeneity of the students studying in N.W.F.P., with the vast majority being indigenous pathans. Karachi however, is a true cosmopolitan city and therefore medical students in Karachi hail from diverse backgrounds like the Punjabi, Sindhi, Pathan and migrant races [24]. Some of them belong to races where smokeless tobacco use is generally believed to be lesser [24]. This is likely to be the contributory factor towards the difference between the rates of usage in N.W.F.P. and Karachi.

Our study also shows a significantly higher prevalence of smoking among users of smokeless tobacco. This could be because the same risk factors probably encourage people to take up smoking as well as the use of smokeless tobacco. In our study, the mean age at which the students started smoking was similar to that at which the students began using smokeless tobacco. This means that both habits are acquired at an equal age, again signifying possible similar reasons behind the use of smoked and smokeless tobacco.

Our finding that the use of smokeless tobacco was more common among the male gender is in line with what was found by Mazahir et al [24]. We feel this is because the use of tobacco remains socially more acceptable for males than females.

On univariate analysis, we found an association between living in the hostel (boarders) and using smokeless tobacco. However, multivariate analysis showed that this was not an independent association, but was likely to be seen because of two major factors. Firstly, most of the boarders were male students in whom the use of smokeless tobacco was much more than female students who made a smaller proportion of the boarders. Secondly, we found a greater number of boarders in N.W.F.P. than in Karachi, that itself being an independent association with the use of smokeless tobacco.

### Limitations

The sample of students used this study is not the perfect representative of Pakistani medical students. This is because we have used convenience sampling as our sampling methodology and our study focuses solely on medical students from three medical colleges of Pakistan although an effort has been made to minimize that bias by selecting colleges from two contrasting regions.

## Conclusion

Use of smokeless tobacco by medical students, although not of alarming proportions, cannot be ignored keeping in mind their future role as care givers. The government should discourage the use of tobacco products as a whole rather than just focusing on cigarette smoking and should realize that as a first step it may not have to begin separate high budget drives against smokeless tobacco. Rather, adding the goal of eliminating smokeless tobacco to existing drives against cigarette smoking may be enough. This is because similar factors seem to be promoting the use of cigarettes as well as smokeless tobacco. Also, medical colleges should consider providing greater education about the myths and hazards of smokeless tobacco. Furthermore, regional preferences for the forms of smokeless tobacco should be kept in mind while planning preventive programmes. Drives in Karachi should focus more on eliminating paan usage while those in NWFP should focus more on the use of naswar. Further community-based studies are required to highlight the health burden due to smokeless tobacco and to better plan anti-tobacco drives in the existing resources of a developing third world country like Pakistan.

## Glossary of terms

*paan/betel quid: *(tobacco added to a mixture of the *Piper betel *leaf, aqueous calcium hydroxide paste [slaked lime], pieces of areca nut [*supari*], and frequently some spices) being the most common one used.

*Naswar: *(a mixture of flavoured tobacco, slaked lime and indigo).

*Gutka: *(a dry preparation of areca nut, slaked lime, catechu, condiments and powdered tobacco).

*Qiwam: *(pellets or thick paste of boiled tobacco mixed with powdered spices),

*Minpuri: *(tobacco with finely cut areca nut, camphor and cloves).

## Competing interests

The author(s) declare that they have no competing interests.

## Authors' contributions

SZI did the overall supervision and participated in the conception of the idea, preparation of the questionnaire and protocol, and writing the manuscript. HN was involved in the overall supervision, preparation of the questionnaire and collection and analysis of data. YJS did the data collection and analysis and was involved in writing the manuscript. AHP designed the study and participated in the preparation of the protocol, and collection and analysis of data. MI participated in the conception of the idea, preparation of the questionnaire and data analysis. SG was involved in the designing the study and writing the manuscript. All authors read and approved the final manuscript.

## Pre-publication history

The pre-publication history for this paper can be accessed here:


